# Mohs Micrographic Surgery Versus Standard Excision for Basal Cell Carcinoma in the Head and Neck: Systematic Review and Meta-Analysis

**DOI:** 10.7759/cureus.19981

**Published:** 2021-11-29

**Authors:** Abdulmalik Alsaif, Amrit Hayre, Mohammad Karam, Shafiq Rahman, Zabihullah Abdul, Paolo Matteucci

**Affiliations:** 1 School of Medicine, University of Leeds, Leeds, GBR; 2 Department of Plastic Surgery, Hull University Teaching Hospitals, Hull, GBR; 3 Department of Plastic Surgery, Nottingham City Hospital, Nottingham, GBR

**Keywords:** plastic and reconstructive surgery, dermatology, surgical excision, standard excision, mohs micrographic surgery, basal cell carcinoma

## Abstract

The objective of this study was to quantitatively compare outcomes between standard excision (SE) and Mohs micrographic surgery (MMS) for basal cell carcinoma (BCC). A systematic review and meta-analysis were performed as per the Preferred Reporting Items for Systematic Reviews and Meta-analyses (PRISMA) Guidelines and a search of electronic databases was conducted to identify all randomised controlled trials (RCTs) and observational studies comparing the outcomes of SE versus MMS for BCC. The primary outcome was the recurrence rate for primary and recurrent BCC. The secondary outcomes included the cost of treatment, aesthetic results, the rate of incomplete excision, and the surgical defect size post excision. Five studies enrolling 2060 lesions were identified. There was a statistically significant difference between MMS and SE groups in terms of recurrence rate for primary BCCs (odds ratio (OR) = 0.44, confidence interval (CI) = 0.16 to 0.97, P = 0.04) and recurrent BCCs (OR = 0.33, CI = 0.12 to 0.97, P = 0.04). For secondary outcomes, MMS had improved results compared with SE, except for mean cost. In conclusion, both primary and secondary BCCs treated with MMS have a reduced recurrence rate and defect size thus simplifying reconstruction. However, due to higher costs and operative time attributed to MMS, it should be reserved for high-risk BCCs.

## Introduction and background

Basal cell carcinoma (BCC) is the most common type of skin cancer across most parts of the world and the single leading cause of cancer among Caucasians [1]. BCCs are associated with risk factors such as age, smoking, ultraviolet radiation, and basal naevus syndrome [2]. Lesions that occur within the H-zone have shown to be at greater risk of recurrence [3]. Although the mortality from BCCs is exceptionally rare due to their small rate of metastases, morbidity may be high if left untreated as they can be locally invasive, causing extensive destruction of the surrounding tissues [4]. The standard treatment for BCCs is surgical excision with an adequate tissue margin, however, there are many modalities that can be used including cryosurgery, curettage, electrodesiccation, radiotherapy, and photodynamic therapy, although these tend to be reserved for low-risk BCCs. These techniques although useful are limited as they do not offer histological confirmation of clearance [5]. The difference between the two techniques is how the lesion is excised in a staged manner, with the difference being that there is tissue sparing in MMS [6]. There are currently two main techniques used for BCC excision, this includes standard excision (SE) and Mohs micrographic surgery (MMS) [5]. The difference between the two techniques is how the lesion is excised in a staged manner, with the difference being that there is tissue sparing in MMS. After the lesion has been excised using SE, the specimen is sliced vertically, like a loaf of bread, and analysed [7]. This only gives an account of less than 2% of the specimen’s margin, however [7]. In MMS, the specimen is sliced horizontally, which captures 100% of the BCC margin. Obtaining greater accuracy of the extent of the lesion’s margins provides better surgical clearance thus leading to lower recurrence rates [8]. Marzuka et al. also found BCCs treated with MMS to result in smaller surgical defects when compared to SE, which can reduce the complexity of reconstruction required [5]. Outcomes for BCCs treated by SE and MMS in the head and neck have been compared in randomised controlled trials (RCTs) as well as observational studies including the rate of carcinoma recurrence [5,9-13]. There are, however, currently no meta-analyses in the literature to quantitively compare outcomes between these two surgical options. This study will be the first in the literature to report on this subject.

## Review

Methods

A systematic review and meta-analysis were performed in accordance with the Preferred Reporting Items for Systematic Reviews and Meta-Analyses (PRISMA) guidelines [12].

Eligibility Criteria

The eligibility criteria included all prospective randomised trials directly comparing excision of BCCs through MMS and SE of the head and neck. Observational studies that had a control group and an intervention group were also included. Studies not reported in English, and those in which other treatment modalities for the management of BCCs were used have been excluded. MMS was the intervention group of interest and SE was the comparator.

Outcome Measures

The primary outcome was the recurrence rate for primary and recurrent BCC. Recurrence was determined by clinical diagnosis with histological confirmation. The secondary outcomes included the cost of treatment, aesthetic results, the rate of incomplete excision, and the surgical defect size post excision.

Literature Search Strategy

Three authors (AA, AH, and MK) searched the following electronic databases: MEDLINE, EMBASE, EMCARE, CINAHL, and the Cochrane Central Register of Controlled Trials (CENTRAL). The last search was run on November 10, 2021. Additionally, thesaurus headings, search operators, and limits in each of the databases were adjusted accordingly. The authors also searched the following websites for details of currently ongoing and unpublished studies: World Health Organization (WHO), International Clinical Trials Registry, ClinicalTrials.gov, and ISRCTN Register. There were no language restrictions applied in our search strategies. The search terminologies included ‘basal cell carcinoma’, ‘BCC’, ‘Mohs micrographic surgery’, ‘MMS’, ‘wide local excision’, and ‘excision’. 

Selection of Studies

The authors assessed the titles and abstracts of articles identified from the literature search. The full texts of relevant studies were read and those that met the eligibility criteria of the current review were selected.

Data Extraction and Management

An electronic data extraction spreadsheet was created in line with Cochrane's data collection form for intervention reviews. The spreadsheet included the following data: first author, year of publication, country of origin of the corresponding author, journal in which the study was published, study design, study size, type of intervention (MMS or SE), patient group (primary BCC and recurrent BCC), baseline demographics of the included populations (age and gender), primary and secondary outcome data.

Data Synthesis

Review Manager 5.3 software (The Nordic Cochrane Centre, The Cochrane Collaboration, Copenhagen) was used for data synthesis. Three authors (AA, AH, and MK) have independently entered the extracted data into the software and used the fixed-effect model to perform the analysis. The results were reported in forest plots with 95% confidence intervals (CIs). For dichotomous outcome variables, the odds ratio (OR) was used as the summary measure. The OR is the odds of an event in the MMS group compared with the SE group. An OR of less than 1 for the recurrence rate would favour the MMS group, and an OR of more than 1 would suggest recurrence is more strongly related to SE.

Assessment of Heterogeneity

The Cochran Q test (χ^2^) was used to assess heterogeneity between the studies. Inconsistency was quantified by calculating I2 and the following guide was used for interpretation: 0% to 25% may represent low heterogeneity, 25% to 75% may represent moderate heterogeneity and 75% to 100% may represent high heterogeneity.

Methodological Quality and Risk of Bias Assessment

Three authors (AA, AH, and MK) independently assessed the methodological quality as well as the risk of bias for articles matching the inclusion criteria. The Newcastle-Ottawa Scale [14] was used for the assessment of bias of observational studies in terms of three domains: selection, comparability, and exposure. It uses a star scoring system with a maximum total score of nine stars for each study. Studies with a score of 9 are considered low risk of bias whereas 6 or lower are an indicator of a high risk of bias.

Results

Literature Search Results

The search strategy retrieved 403 studies, and after a thorough screening of the retrieved articles, the authors identified six studies in total which met the eligibility criteria (Table 1).

**Table 1:** Search results of databases. Search results of databases that identified six studies meeting the eligibility criteria [7,9-13].

Database	No. of articles identified
PubMed	67
EMBASE	107
MEDLINE	65
EMCARE	9
CINAHIL	6
Google Scholar	147
Additional articles identified through bibliographic searches	2
Articles excluded	Duplicates: 148, non-comparative: 160, Not related to head and neck: 89

Primary Outcomes

**Figure 1:**
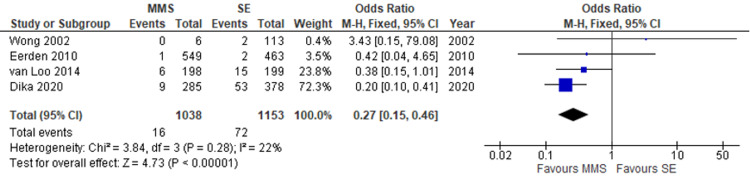
Recurrence rate of primary BCCs treated by MMS and SE of the head and neck. Forest plot for of four studies [9,11-13] showing recurrence rate of primary basal cell carcinomas (BCCs) of the head and neck treated by Mohs micrographic surgery (MMS) and standard excision (SE). Odds ratio analyses showing a significantly lower recurrence rate for the MMS cohort.

Recurrence rate: Recurrence rates for primary BCCs were reported by four studies enrolling 2191 lesions in total (Figure 1). There was a statistically significant difference seen in the odds ratios analysis (OR) showing significantly fewer recurrence rates in primary lesions treated by MMS (OR= 0.27, CI = 0.15 to 0.46, P ≤ 0.00001). A low level of heterogeneity was found amongst the studies giving consistency to the outcome (I2 = 22%, P = 0.28).

**Figure 2:**
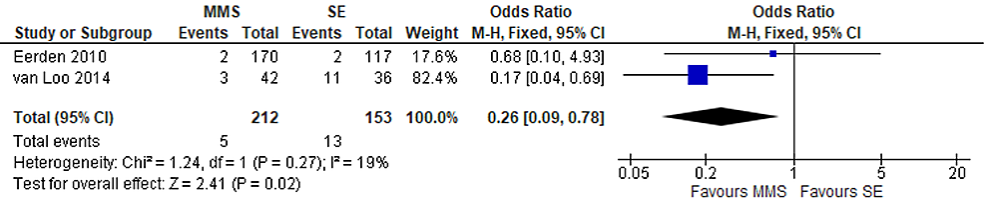
Recurrence rate of recurrent BCCs treated by MMS and SE of the head and neck. Forest plot of two studies [9,11] showing recurrence rate of recurrent BCCs of the head and neck treated by Mohs micrographic surgery (MMS) and standard excision (SE). Odds ratio analyses showing a significantly lower recurrence rate for the MMS cohort.

The recurrence rate for recurrent BCCs was reported in two studies enrolling 365 lesions (Figure 2). There was a statistically significant difference seen in the odds ratio analyses showing a lower recurrence rate for lesions treated by MMS (OR = 0.26, CI = 0.09 to 0.78, P = 0.02). A low level of heterogeneity was found amongst the studies (I2 = 19%, P = 0.27).

All three included studies followed up their patients at different time periods, varying from 16 months to 10 years (Table 2). Van loo et al. [9] offered the longest follow-up period at 10 years. Although Van der Eerden et al. [11] assessed patients at a mean follow up of 2.28 years and 3.14 years in the SE and MMS group, respectively, there were still a significant proportion of patients who were followed up for over five years (20% for MMS and 12% for SE). Similarly, Wong et al. [12] reported that 97 BCC lesions had follow-ups of at least five years although their mean rate was lower (1.6 years).

**Table 2:** Follow-up periods for basal cell carcinoma lesions of included studies in quantitative analysis. BCC: basal cell carcinoma, SE: standard excision, MMS: Mohs micrographic surgery.

Study	BCC Lesion	Intervention	Follow-up period (months/years)
Van Loo et al. [9]	Primary and recurrent	SE	120 m/10 yrs
	Primary and recurrent	MMS	120 m/10 yrs
Van der Eerden et al. [11]	Primary and recurrent	SE	27.4 m/2.28 yrs (mean)
	Primary and recurrent	MMS	37.7 m/3.14 yrs (mean)
Wong et al. [12]	Primary	SE	31.2 m/2.6 yrs (mean)
	Primary	MMS	20.4 m/1.6 yrs (mean)

A summary of the recurrences for all BCCs is given in Table 3. The mean time to recurrence appeared to be shorter in the recurrent BCC subgroup, which could be attributed to greater intensity of follow-up based on the authors’ own clinical experience, however, information relating to this is not provided by the articles analysed.

**Table 3:** Summary of all basal cell carcinoma recurrences; histological subtype, mean time to recurrence, and anatomical location of head and neck region. BCC: basal cell carcinoma, SE: standard excision, MMS: Mohs micrographic surgery.

	Study	Total no. of recurrences	Histological subtype	Mean time to recurrence (months)	Location	Intervention: SE/MMS
Primary BCCs	Van loo et al. [9]	25	Aggressive: 6, non-aggressive: 19	68.60	Frontal/ temporal: 14, perinasal: 9, ear: 2	SE: 17, MMS: 8
	Van der Eerden et al. [11]	3	Aggressive: 2, non-aggressive: 1	85.33	Nose: 2, ear: 1	SE: 2. MMS: 1
	Wong et al. [12]	2	NR	NR	Peri-ocular: 2	SE: 2, MMS: 0
	Dika et al. [13]	62	Reported that MMS recurrences were of an aggressive type	NR	Nose area in the MMS group	SE: 53, MMS: 9
Recurrent BCCs	Van Loo et al. [9]	14	Aggressive: 4, non-aggressive: 10	38.30	Frontal/ temporal: 6, perinasal: 4, cheek/chin: 2, periocular: 2	SE: 11, MMS: 3
	Van der Eerden et al. [11]	4	Aggressive: 4, non-aggressive: 0	No data for 2 cases. Mean time for 2 reported: 42.50.	Nose: 2, ear: 1, lip/chin: 1	SE: 2, MMS: 2

Secondary Outcomes

Mean cost: The operative costs of MMS and SE were reported only by Smeets et al. [10] and the authors took into consideration the staff involved in the procedure, materials used, and the histopathological processing and examining of slides. According to Smeets et al. [10], MMS had a higher total operative cost than SE in removing BCC of both primary and recurrent origin (p<0.001).

Aesthetic results: Smeets et al. [10] involved the patients to judge the aesthetic result at 6 and 18 months post-operatively. Photographs of selected BCCS (first 139 primaries, first 89 recurrent) were judged retrospectively also by three professionals and three laymen. Using the Pearson product-moment correlation coefficient, Smeets et al. concluded that there was no significant difference in the aesthetic outcomes between MMS and SE-treated BCC lesions. However, they found that primary tumours had a significantly better aesthetic outcome compared to recurrent tumours (p=0.038) regardless of the technique of excision.

Incomplete excision: Van loo et al. [9], Smeets et al. [10], and Van der Eerden et al. [11] looked into the rate of incomplete excision following SE for both primary and recurrent BCC. Overall, the studies showed that recurrent BCCs had a higher rate of incomplete excision than primary BCC following SE (Table 4).

**Table 4:** Rates of incomplete excision for standard excision for primary and recurrent basal cell carcinoma. BCC: basal cell carcinoma, SE: standard excision.

	Van loo et al. [9]	Smeets et al. [10]	Muller et al. [7]	Van der Eerden et al. [11]	Wong et al. [12]
Primary BCC	18%	18%	NR	18%	NR
Recurrent BCC	32%	32%	NR	30%	NR

Surgical defect size: Smeets et al. [10] calculated the mean defect size post excision and no significant difference was found between SE and MMS for both primary and recurrent BCCs; however, a significantly smaller defect size was obtained for those lesions needing more than one SE for clearance or two stages of MMS. Muller et al. [7] also calculated the surgical defect size and found that lesions treated with MMS had a significantly smaller median defect size of 116.2mm^2^, compared to 187.7mm^2^ in the SE intervention group (95% CI for difference = 61-126, p<0.001).

All studies within the review reported on a range of histological subtypes for BCCs consisting primarily of aggressive and non-aggressive type forms as summarised in Table 5.

**Table 5:** Histological subtype and anatomical distribution of basal cell carcinomas across selected studies. MMS: Mohs micrographic surgery, SE: standard excision, NR: not reported.

Study (year)	Histological subtypes		
	Aggressive	Non-aggressive	Unknown
Van Loo et al. [9] and Smeets et al.[10]	MMS 165, SE 137	MMS 137, SE 168	MMS, 4 SE 1
Van der Eerden et al. [11]	MMS 349, SE 210	MMS 370, SE 370	NR
Muller et al. [7]	NR	MMS 12, SE 14	NR
Wong et al. [12]	Total 227, infiltrating: 123, morpheic: 35, pigmented: 6, basosquamous: 16, micronodular: 47	Total 545, solid: 450, superficial: 80, adenoid: 15	57
Dika et al. [13]	MMS 272, SE 276	MMS 13, SE 102	NR

Table 6 below summarises the baseline characteristics of the included studies. 

**Table 6:** Baseline characteristics of the included studies. MMS: Mohs micrographic surgery, SE: standard excision, RCT: randomised control trial, SD: standard deviation.

Study (year)	Journal, country	Age (years)	Sex: (male/female)	Study design	Total number of lesions	Interventions compared
Van Loo et al. [9]	European Journal of Cancer, Netherlands	Primary: 67.7 ± 12.65 (mean ± SD); recurrent: 67.9 ± 11.7 (mean ± SD)	Total - 334:231, primary - 224:150, recurrent - 110:81	RCT	Primary: 204 vs. 204, recurrent: 102 vs. 102	MMS vs. SE
Smeets et al. [10]	The Lancet, Netherlands	Primary: 67.7 ± 12.65 (mean ± SD); recurrent: 67.9 ± 11.7 (mean ± SD)	Total - 334:231, primary - 224:150, recurrent - 110:81	RCT	Primary: 204 vs. 204, recurrent: 102 vs. 102	MMS vs. SE
Van der Eerden et al. [11]	Laryngoscope, Netherlands	73 (median) MMS vs. SE: 58.5 vs. 73 (median)	Total - 791:713, MMS - 371:424, SE - 421:288	Observational	Primary: 549 vs. 463, recurrent: 170 vs. 117	MMS vs. SE
Muller et al. [7]	American Society for Dermatologic Surgery, United Kingdom	MMS vs. SE: 66 vs. 72 (mean)	Not reported	RCT	Primary: 15 vs. 15, recurrent - 0	MMS vs. SE
Wong et al. [12]	American Society of Ophthalmic Plastic and Reconstructive Surgery, Inc., Australia	Total: 64 ± 16 (mean ± SD)	Total - 317:302	Observational study	Total: 113 vs. 6	MMS vs. SE (the frozen section was also compared but authors excluded this data)
Dika et al. [13]	Dermatologic therapy, Italy	NR	NR	Retrospective cohort study	Total: 663, MMS: 285, SE: 378	MMS vs. SE

Methodological Quality and Risk of Bias Assessment

The Cochrane Collaboration tool was used to assess the quality of the RCTs included in the study (Table 7). The Newcastle-Ottawa scale [14] was used to assess the quality of the non-randomised studies (Table 8). All studies involved showed a high quality for selection and exposure domains.

**Table 7:** Bias analysis of the randomised trials using the Cochrane Collaboration’s tool.

Study	Bias	Authors’ judgement	Support for judgement
Van Loo et al. [9]	Random sequence generation (selection bias)	Low risk	A computer-generated allocation scheme (Sampsize 2.0) was used and randomisation via telephone by an independent person not involved in the trial.
	Allocation concealment (selection bias)	Unclear risk	No information was given.
	Blinding of participants and personnel (performance bias)	High risk	For practical reasons, no blinding was performed for the allocated treatment.
	Blinding of outcome assessment (detection bias)	Unclear risk	No information was given.
	Incomplete outcome data (attrition bias)	High risk	Only 35-40% of patients completed 10 years follow-up.
	Selective reporting (reporting bias)	Low risk	All outcome data reported.
	Other bias	Low risk	Similar baseline characteristics in both groups.
Smeets et al. [10]	Random sequence generation (selection bias)	Low risk	A computer-generated allocation scheme (Sampsize 2.0) was used and randomisation by an independent person not involved in the trial.
	Allocation concealment (selection bias)	Unclear risk	The paper has written the research physician allocated patients to either MMS or SE but it is not documented how the allocation choice was made.
	Blinding of participants and personnel (performance bias)	High risk	For practical reasons, no blinding was performed for the allocated treatment.
	Blinding of outcome assessment (detection bias)	Unclear risk	No information was given.
	Incomplete outcome data (attrition bias)	Low risk	Although the number of BCC lesions studied dropped by 77 from the originally allocated number, each drop out was documented for and justified.
	Selective reporting (reporting bias)	Low risk	All outcome data reported.
	Other bias	Low risk	Similar baseline characteristics in both groups.
Muller et al. [7]	Random sequence generation (selection bias)	Low risk	A randomization procedure was done using opaque-sealed envelopes containing the words “Mohs” or “Standard” to allocate patients.
	Allocation concealment (selection bias)	Unclear risk	No information was given.
	Blinding of participants and personnel (performance bias)	High risk	For practical reasons, no blinding was performed for the allocated treatment.
	Blinding of outcome assessment (detection bias)	Unclear risk	No information was given.
	Incomplete outcome data (attrition bias)	Low risk	Although the number of participants decreased from the originally allocated number, each dropout was documented for and justified.
	Selective reporting (reporting bias)	Low risk	All outcome data reported.
	Other bias	Low risk	Similar baseline characteristics in both groups.

**Table 8:** Newcastle-Ottawa scale to assess the quality of the included observational studies.

Study	Selection	Comparability	Exposure
Van der Eerden et al. [11]	***	*	**
Wong et al. [12]	***	*	**
Dika et al. [13]	***	**	**

Discussion

The results of this meta-analysis indicate that MMS is a superior option to SE in terms of reducing the size of the surgical defect, lowering the recurrence rate as well as offering completeness of excision in a single stage; however, it does not take into account the cost, time or resources required. MMS has certainly been shown to reduce the rate of recurrence for both primary and recurrent BCCs of the head and neck region. An odds ratio analysis for both sub-types reported a low heterogeneity giving consistency to this outcome. Although the follow-up periods were varied amongst the individual studies with Van Loo et al. [9] reporting the longest of 10 years, Van der Eerden et al. [11] and Wong et al. [12] still had a significant proportion of patients who were followed up for over five years. MMS can minimise the size of the surgical defect. Smeets et al. [10] and Muller et al. [7] both reported statistically significant differences in the size of the wounds created post excision with MMS. This can therefore simplify the reconstructive procedure necessitated restoring local anatomy. Incomplete excision rates for SE have been reported by Van Loo et al. [9] and Van der Eerden et al. [11] with both demonstrating a higher rate for recurrent BCCs. Van Loo et al. [9] reported 32% for recurrent BCCs and 18% only for primary BCCs. Van der Eerden et al. [11] reported an incomplete excision rate of 18% for primary BCCs and 30% for recurrent BCCs with SE. Recurrent BCC lesions are therefore a high-risk indicator of incomplete excision and advocate the use of MMS to offer a higher probability of clearance. This has been previously suggested by Telfer et al. [6]. Smeets et al. [10] reported on the overall aesthetic outcome to show no differences between either MMS or SE but found that for primary BCCs the result was superior compared to recurrent ones. The cosmetic appearance was also found to become more inferior with the increasing size of primary BCCs.

The cost of MMS is shown to be significantly more by Smeets et al. [10] when compared to SE and therefore advocates judicious use given financial constraints on many health services. High-risk indicators for incomplete excision by SE such as recurrent BCCs [5] or aggressive histological subtypes should be considered when selecting MMS so as to best optimise available resources. It is important to note that the operative time for MMS is longer than for SE, suggesting SE is still required for lower risk BCCs in areas where there is a high demand for BCC excision [15]. Essers et al. [16] have also reported a significantly higher cost for facial BCC excision compared to SE with 254 euros more for every primary BCC excised by MMS compared to SE. They have, therefore, advocated against its use on any large-scale basis due to not being cost-effective.

Inherent limitations of this review should be accounted for when interpreting the results as excision margins were inconsistent across all studies. Van der Eerden et al. [11] reported a SE margin of between 3 and 5 mm, Wong et al. [12] reported the use of 2 mm for small nodular BCCs and 4 mm for larger ill-defined BCCs. Van Loo et al. [9] on the other hand used a 3 mm standard excision margin compared to the 4 mm used by Muller et al. [7]. In addition, the studies involved BCCs of different morphologies and locations, which have been outlined in Tables 5 and 9. Only one RCT by Van Loo et al. [9] reported on BCC recurrence rates long term as the other studies were observational only and the report by Smeets et al. [10] was the same RCT as Van Loo et al. [9] but with different follow-up periods. In addition, Van Loo et al. [9] also reported that only between 35% and 40% of patients completed a ten-year follow-up. The authors, therefore, suggest the need for more high-quality RCTs with longer follow-up periods to further the current evidence base. The time period for recurrences in primary BCCs has been shown by Van Loo et al. [9] and Van der Eerden et al. [11] to occur after the first five years and therefore emphasises the need for long-term evaluation of both treatment modalities.

**Table 9:** Anatomical distribution of basal cell carcinoma lesions in head and neck region. BCC: basal cell carcinoma, MMS: Mohs micrographic surgery, SE: standard excision, NR: not reported.

Anatomical location							
Study (year)	Lips	Ears	Peri-ocular	Cheek	Nasal/perinasal	Frontal/temporal	Others
Van Loo et al. [9] and Smeets et al. [10]	MMS 20, SE 9	MMS 97, SE134	MMS 76, SE 53	MMS SE	MMS 481, SE 220	MMS 91, SE 111	Peri-auricular: MMS 33, SE 28
Van der Eerden et al. [11]	MMS 59, SE 48	MMS 97, SE134	MMS 76, SE 53	MMS 29, SE 86	MMS 481, SE 220	MMS 46, SE 158	Neck: MMS 7, SE 9
Muller et al. [7]	NR	NR	NR	NR	NR	NR	Head and neck: MMS 12, SE 14
Wong et al. [12]	NR	NR	MMS 6, SE 113	NR	NR	NR	NR
Dika et al. [13]	NR	MMS 20, SE 15	MMS 29, SE 8	MMS 23, SE 177	MMS 160, SE 53	MMS 29, SE 64	Scalp MMS 4, SE 23

## Conclusions

The authors report the first meta-analyses in the literature comparing MMS versus SE in the treatment of BCCs within the head and neck region. MMS reduces the recurrence rate and defect size thus simplifying reconstruction but advocates judicious use given the higher cost and should therefore be reserved for more high-risk BCCs. The authors suggest that further high-quality RCTs are conducted with long-term outcomes to improve the current database and so better guide clinicians on the optimum treatment.

## References

[1] Lucas R, McMichael T, Smith W, Armstrong BK (2021). Solar ultraviolet radiation: global burden of disease from solar ultraviolet radiation. https://apps.who.int/iris/handle/10665/43505.

[2] Raasch BA, Buettner PG, Garbe C (2006). Basal cell carcinoma: histological classification and body-site distribution. Br J Dermatol.

[3] Rigel DS (2008). Cutaneous ultraviolet exposure and its relationship to the development of skin cancer. J Am Acad Dermatol.

[4] Swanson NA (19831). Mohs surgery: technique, indications, applications, and the future. Arch Dermatol.

[5] Marzuka AG, Book SE (2015). Basal cell carcinoma: pathogenesis, epidemiology, clinical features, diagnosis, histopathology, and management. Yale J Biol Med.

[6] Telfer NR, Colver GB, Morton CA (2008). Guidelines for the management of basal cell carcinoma. Br J Dermatol.

[7] Muller FM, Dawe RS, Moseley H, Fleming CJ (2009). Randomized comparison of Mohs micrographic surgery and surgical excision for small nodular basal cell carcinoma: tissue-sparing outcome. Dermatol Surg.

[8] Narayanan K, Hadid OH, Barnes EA (2012). Mohs micrographic surgery versus surgical excision for periocular basal cell carcinoma. Cochrane Database Syst Rev.

[9] van Loo E, Mosterd K, Krekels GA (2014). Surgical excision versus Mohs' micrographic surgery for basal cell carcinoma of the face: a randomised clinical trial with 10 year follow-up. Eur J Cancer.

[10] Smeets NW, Krekels GA, Ostertag JU, Essers BA, Dirksen CD, Nieman FH, Neumann HM (2004). Surgical excision vs Mohs' micrographic surgery for basal-cell carcinoma of the face: randomised controlled trial. Lancet.

[11] van der Eerden PA, Prins ME, Lohuis PJ, Balm FA, Vuyk HD (2010). Eighteen years of experience in Mohs micrographic surgery and conventional excision for nonmelanoma skin cancer treated by a single facial plastic surgeon and pathologist. Laryngoscope.

[12] Wong VA, Marshall JA, Whitehead KJ, Williamson RM, Sullivan TJ (2002). Management of periocular basal cell carcinoma with modified en face frozen section controlled excision. Ophthalmic Plast Reconstr Surg.

[13] Moher D, Liberati A, Tetzlaff J, Altman DG (2009). Preferred reporting items for systematic reviews and meta-analyses: the PRISMA statement. Ann Intern Med.

[14] Dika E, Veronesi G, Patrizi A (2020). It's time for Mohs: micrographic surgery for the treatment of high-risk basal cell carcinomas of the head and neck regions. Dermatol Ther.

[15] Wells GA, Shea B, O'Connell D (2021). The Newcastle-Ottawa Scale (NOS) for assessing the quality of nonrandomized studies in meta-analyses. http://www.ohri.ca/programs/clinical_epidemiology/oxford.asp.

[16] Essers BA, Dirksen CD, Nieman FH, Smeets NW, Krekels GA, Prins MH, Neumann HA (2006). Cost-effectiveness of Mohs micrographic surgery vs surgical excision for basal cell carcinoma of the face. Arch Dermatol.

